# Association between combined healthy lifestyles and rheumatoid arthritis: A cross-sectional study based on the NHANES database

**DOI:** 10.1097/MD.0000000000045482

**Published:** 2025-11-07

**Authors:** Shouyu Miao, Xuming Zhang

**Affiliations:** a Department of Rheumatology and Immunology, The First Hospital of Jilin University, Changchun, China; b Department of Urology, Beijing Tsinghua Changgung Hospital, School of Clinical Medicine, Tsinghua Medicine, Tsinghua University, Beijing, China.

**Keywords:** healthy lifestyle, NHANES, rheumatoid, smoking, waist circumference

## Abstract

The aim of our research was to explore the relationship between healthy lifestyles and rheumatoid arthritis (RA). Data were collected from the National Health and Nutrition Examination Survey database from 1999 to 2018. The healthy lifestyle score (ranging from 0 to 5) was assessed based on current nonsmoking, low to moderate alcohol drinking, adequate physical activity, optimal waist circumference, and healthy diet. RA patients were identified from the questionnaire data. Weighted multiple regression analysis and subgroup analysis were employed to investigate the association between healthy lifestyles and RA. On this basis, a restricted cubic spline plot was used to examine the nonlinear relationship. A total of 25,325 patients were included in our study, among whom 1236 had RA. After adjusting for all covariates, the multivariable logistic regression analysis indicated that participants adhering to 4 to 5 healthy lifestyle behaviors had a lower probability of having RA compared to those adhering to 0 to 1 healthy lifestyle behaviors (odds ratio = 0.417, 95% confidence interval = 0.304–0.573, *P* < .001). The interaction test revealed an interaction effect of age. The weighted healthy lifestyle score was more effective in assessing the association between lifestyle and the presence of RA, and the restricted cubic spline plot demonstrated a linear relationship. Overall, adherence to a greater variety of healthy lifestyle behaviors shows a negative association with the occurrence of RA.

## 1. Introduction

Rheumatoid arthritis (RA) is a chronic inflammatory disorder characterized by systemic autoimmune diseases and inflammatory synovitis, affecting 0.5% to 1.0% of the global population.^[1–3]^ It is characterized by joint pain, swelling and stiffness, resulting in a decline in mobility and a reduction in the quality of life and this disease can also affect various organs and systems beyond the joints, including the lungs, heart, kidneys, eyes, skin, and nervous system.^[4,5]^ It has been reported that absenteeism and work disability related to RA account for at least 39%, resulting in a substantial economic impact.^[6]^ The occurrence and development of RA are highly complex and the outcome of the interaction of multiple factors, being closely associated with genetic and environmental factors.^[7–9]^ Previous studies have shown that genetic factors account for 50% to 60% of the risk of RA, while the rest may be influenced by environmental factors.^[10,11]^ Environmental factors such as smoking and dietary choices exert a vital role in the management of the disease. Smoking is associated with the occurrence and disease progression of RA, while certain dietary selections, for instance, avoiding the intake of red meat and integrating more fruits and oily fish into the diet, might contribute to reducing the risk of RA.^[12,13]^ Contrary to prevalent assumptions, low to moderate alcohol consumption may exhibit a negative association with the progression of RA.^[14]^

In recent years, healthy lifestyles have garnered escalating attention and focus, as they can enhance individual health by means of low-cost alterations in life behaviors.^[15–17]^ A healthy lifestyle score can be composed of the following factors: current nonsmoking, low to moderate alcohol drinking, adequate physical activity, optimal waist circumference, and healthy diet.^[18]^ A healthy lifestyle can lower the risk of multiple diseases, such as periodontitis, nonalcoholic fatty liver disease, diabetes, cardiovascular diseases, chronic respiratory disorders, and certain malignancies.^[19–21]^ Numerous studies have demonstrated that single-factor lifestyle modifications can influence the occurrence and development of RA,^[22–24]^ however, few studies have uncovered the relationship between comprehensive lifestyles and RA. A study from Iran pointed out that individuals who choose a healthier lifestyle have a lower risk of developing RA compared to those with a less healthy lifestyle. However, this study was conducted in Dena County (Sisakht region) near Yasuj city, Iran. Another study based on the UK Biobank also found a significant association between the 2,^[25]^ and there have been no large-scale studies based on the US National Health and Nutrition Examination Survey (NHANES) database yet.^[26]^

Although many studies support that various single-factor lifestyles promote the occurrence of RA, the chronic inflammation and pain burden of RA patients may also have changed their lifestyles. The occurrence of RA may make it increasingly difficult to maintain a healthy lifestyle, the pain caused by RA may lead to a reduction in the patient’s physical activity,^[27]^ smoking, drinking alcohol and a high-sugar diet may help patients relieve pain, anxiety or the burden of disease caused by their illness.^[28–30]^ The use of glucocorticoids, one of the therapeutic drugs, may also lead to an increase in waist circumference.^[31]^

Consequently, the objective of our research was to employ the data from the NHANES to investigate the association between healthy lifestyle scores and RA. This work aims to provide observational evidence regarding the link between healthy lifestyles and RA prevalence at a single time point, thereby providing a new direction for evidence-based research on RA.

## 2. Materials and methods

### 2.1. Study population

The NHANES is a nationally representative survey program aimed at evaluating the health and nutrition conditions of adults and children in the United States. Since 1999, this survey has been conducted biennially, recruiting stratified, multistage, and probability-based aggregated samples in 15 counties nationwide, with approximately 5000 participants. The survey encompasses the collection of demographic, socioeconomic, and lifestyle information through household interviews; subsequently, a physical examination is conducted, including dietary interviews, medical and physical measurements, as well as laboratory tests carried out by trained medical staff at Mobile Examination Centers (MEC). Detailed information regarding the research design, recruitment procedures, and data collection can be obtained from the Centers for Disease Control and Prevention. The NHANES protocol was approved by the Research Ethics Review Board of the National Center for Health Statistics, and all participants provided written informed consent.

**Figure 1.**
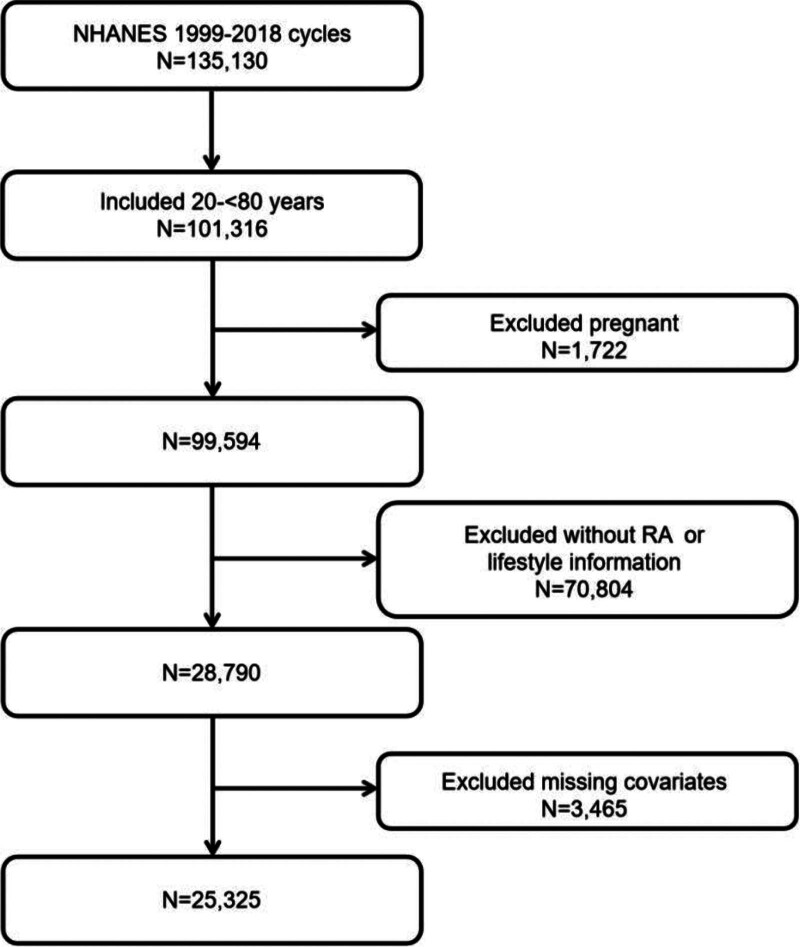
Flow of eligible participants selection. NHANES = National Health and Nutrition Examination Survey, RA = rheumatoid arthritis.

We consolidated the NHANES datasets from 1999 to 2018 across 10 cycles. We included nonpregnant participants aged between 20 and 80 (excluding 80). Among the 99,594 nonpregnant individuals, we excluded those with missing data on arthritis and healthy lifestyle score. Missing data include follow-up loss, questionnaire skips, participant refusals to respond, or lack of awareness. Furthermore, we also excluded participants with missing covariates (Fig. 1). Eventually, 25,325 participants were included.

### 2.2. Construction of healthy lifestyle score

The healthy lifestyle score is derived by summing up the number of healthy lifestyle factors, which comprise being a current nonsmoking, low to moderate alcohol drinking, adequate physical activity, healthy diet, and optimal waist circumference.^[18]^ The healthy lifestyle score ranges from 0 to 5, where a higher score implies a healthier lifestyle. The health level of lifestyle factors is defined as depicted in Table 1. Regarding smoking, current nonsmoking is regarded as the healthy level. With respect to alcohol drinking, the healthy level is defined as low-to-moderate alcohol drinking which indicates a daily alcohol intake of 1 to 28 g for men and 1 to 14 g for women. Regarding physical activity, the healthy level is defined as undertaking more than 150 minutes of moderate-to-vigorous leisure-time physical activity on a weekly basis.^[18,32]^ The healthy eating index-2015 (HEI-2015) comprises 13 components, of which 9 are designated for adequate intake and 4 are recommended to be limited, serving as a tool for evaluating dietary quality.^[33]^ A healthy diet is defined as being within the top three-fifths of the HEI-2015 score. In this research, we employed waist circumference to evaluate obesity, and the healthy level was defined as <94 cm for men and <80 cm for women.

**Table 1:** Definition of a healthy lifestyle.

Factor	Healthy level	Unhealthy level
Tobacco smoking	Current nonsmoking	Current smoking
Alcohol drinking	Men: 1–28 g/d Women: 1–14 g/d	Men: 0 or >28 g/d Women: 0 or >14 g/d
Physical activity	Moderate-to-vigorous leisure-time physical activity of ≥150 min/wk	Moderate-to-vigorous leisure-time physical activity of <150 min/wk
Diet	Top 2-fifths of HEI-2015 score	Bottom three-fifths of HEI-2015 score
Waist circumference	Men: <94 cm Women: <80 cm	Men: ≥94 cm Women: ≥80 cm

HEI = healthy eating index.

### 2.3. Definition of rheumatism arthritis

RA was ascertained with a self-report questionnaire. Specifically, participants were asked that “Has a doctor or other health professional ever told you that you had arthritis?” Those who answered “yes” were then asked a second question, “Which type of arthritis was it?” Participants were classified as RA or not according to their answers to the second question. Participants were regarded as having RA when the first response was “Yes” and the second response was “Rheumatoid arthritis.” A prior study demonstrated that self-reported arthritis has a considerable consistency with clinically confirmed arthritis (85%).^[34]^

### 2.4. Assessment of covariates

Demographic, socioeconomic and lifestyle information was collected by trained interviewers through a computer-assisted personal interview system. Blood pressure data were measured by professionals using mercury sphygmomanometers, and blood glucose and glycated hemoglobin were measured in professional laboratories. Races were classified as non-Hispanic White and other races. Self-reported educational attainment was categorized as below high school, high school, and above high school. The family poverty income ratio (PIR) was calculated by dividing the family (or individual) income by the poverty guideline in the survey year to assess the income status. Hypertension was defined as a self-reported doctor’s diagnosis of hypertension, multiple measurements of systolic blood pressure ≥ 140 mm Hg, diastolic blood pressure ≥ 90 mm Hg, or current use of prescribed drugs for treating hypertension. Diabetes was defined as a self-reported doctor’s diagnosis of diabetes, fasting plasma glucose ≥ 126 mg/dL, glycated hemoglobin A1c ≥ 6.5%, 2-hour blood glucose in the oral glucose tolerance test ≥ 200 mg/dL, and the use of insulin or oral hypoglycemic drugs.

### 2.5. Statistical methods

Data analysis was performed using R (version 4.3.0, http://www.R-project.org). The NHANES database was investigated through a complex and multistage sampling approach. Since our data utilized the data collected by MEC, the MEC examination weights (WTMEC4YR, WTMEC2YR) were employed for the analysis. Continuous variables were presented as weighted means and standard deviations, while categorical variables were presented as weighted percentages. Chi-square tests and *T*-tests were respectively utilized to compare the categorical and continuous variables among different groups.

Weighted logistic regression was employed to investigate the association between the healthy lifestyle score and the risk of RA. As presented in Table 2, the variance inflation factors of all variables were far below the multicollinearity threshold variance inflation factor = 10). In model 1, adjustments were made for age (<50 years, ≥50 years), gender (male, female), and race (non-Hispanic White, others). In model 2, we made further adjustments for PIR (<3.5, ≥3.5), educational attainment (above high school, at or below high school), hypertension (yes, no), and diabetes (yes, no). We also computed the multivariable adjusted odds ratio (OR) and 95% confidence interval (CI) for RA associated with each additional healthy lifestyle factor. To explore whether the aforementioned confounding factors would modify the association between the healthy lifestyle score and the risk of RA, we carried out subgroup analyses and interaction tests. To study the contribution of different lifestyle factors, we initially evaluated the association between 5 lifestyle factors and RA and made mutual adjustments for all lifestyle factors. Subsequently, we removed 1 lifestyle factor from the score each time, reconstructed a new healthy lifestyle score, and adjusted for the removed factor in the model. To further evaluate the robustness of the results, we utilized the *E* value to compute the assessment of potential residual confounding factors. The *E* value is defined as: the minimum strength of association between unmeasured confounding factors and exposure and outcome, conditional on measured covariates, to fully account for the observed exposure-outcome association.^[35,36]^ Finally, we constructed a weighted healthy lifestyle score to better reflect the influence of each healthy lifestyle factor on the outcome. A score of 1 was assigned for a healthy lifestyle, otherwise 0. The weighted and standardized healthy lifestyle score was computed based on the β coefficients of each lifestyle in a logistic regression model encompassing all 5 lifestyle factors and adjusted for all covariates. Each binary lifestyle factor was multiplied by its β coefficient, summed up, divided by the sum of the β coefficients, and then multiplied by 5. The weighted score varies from 0 to 5, comprehensively considering the OR values of each lifestyle. Subsequently, we divided the weighted lifestyle score into quartiles to avoid extreme groups. We further plotted the restricted cubic spline curve to visually demonstrate the dose–response relationship between the weighted healthy lifestyle score and RA.

**Table 2:** The variance inflation factors (VIFs) of each variable.

Variable	VIF
Age	1.33
Sex	1.15
Race	1.23
PIR	1.46
Education	1.45
Hypertension	1.07
Diabetes	1.08
Healthy lifestyle score	1.31

PIR = poverty income ratio, VIF = variance inflation factor.

All statistical tests were conducted using 2-sided tests, and a *P*-value <.05 was regarded as having statistically significance.

This study is based on de-identified data collected from the NHANES and does not contain any experimental data with human or animal participants; participants were not directly involved in the submission, and secondary data were used for this analysis. The NCHS Ethics Review Board approval waived the requirement for informed consent from all patients.

## 3. Results

### 3.1. Population characteristics

This cross-sectional study included 25,352 adult Americans, with a weighted mean age of 47.02 years and 54% male participants. The prevalence rates for current nonsmoking, low-to-moderate alcohol drinking, adequate physical activity, healthy diet, and optimal waist circumference were 76.0%, 70.2%, 43.1%, 39.2%, and 26.0%, respectively. Table 3 illustrates the clinical characteristics of participants stratified by healthy lifestyle score. Notably, significant statistical differences were observed across all variables examined in this study (all *P* < .05).

**Table 3:** The weighted baseline characteristics of the study population.

Variable	Overall	Score of healthy lifestyle				SMD	*P*-value
		0–1	2	3	4–5		
	n = 25,325 N = 1,18,17,75,234	n = 4864 N = 20,02,26,936	n = 7614 N = 34,44,15,984	n = 7342 N = 34,85,50,895	n = 5505 N = 28,85,81,419		
Age (mean [SD])	47.02 (15.37)	49.73 (14.63)	48.25 (15.21)	46.39 (15.62)	43.78 (15.38)	0.204	<.001
Gender (n)%						0.121	<.001
Male	13,684 (54.0)	2392 (49.2)	3950 (51.9)	4030 (54.9)	3312 (60.2)		
Female	11,641 (46.0)	2472 (50.8)	3664 (48.1)	3312 (45.1)	2193 (39.8)		
Race (n)%						0.067	<.001
White	12,293 (48.5)	2450 (50.4)	3533 (46.4)	3448 (47.0)	2862 (52.0)		
Others	13,032 (51.5)	2414 (49.6)	4081 (53.6)	3894 (53.0)	2643 (48.0)		
Education (n)%						0.431	<.001
Under high school	5271 (20.8)	1627 (33.4)	1764 (23.2)	1339 (18.2)	541 (9.8)		
High school	5810 (22.9)	1392 (28.6)	1990 (26.1)	1549 (21.1)	879 (16.0)		
Above high school	14,244 (56.2)	1845 (37.9)	3860 (50.7)	4454 (60.7)	4085 (74.2)		
PIR (n)%						0.413	<.001
<3.5	16,225 (64.1)	3963 (81.5)	5273 (69.3)	4416 (60.1)	2573 (46.7)		
≥3.5	9100 (35.9)	901 (18.5)	2341 (30.7)	2926 (39.9)	2932 (53.3)		
Waist (mean [SD])	98.94 (16.37)	105.40 (15.77)	102.43 (16.34)	97.86 (15.48)	89.84 (13.61)	0.566	<.001
HEI2015 (mean [SD])	52.24 (13.72)	43.36 (9.38)	47.97 (11.68)	54.66 (13.20)	62.76 (11.76)	0.940	<.001
Tobacco smoking (n)%						0.810	<.001
Unhealthy level	6070 (24.0)	2975 (61.2)	1967 (25.8)	947 (12.9)	181 (3.3)		
Healthy level	19,255 (76.0)	1889 (38.8)	5647 (74.2)	6395 (87.1)	5324 (96.7)		
Alcohol drinking (n)%						0.941	<.001
Unhealthy level	7542 (29.8)	3486 (71.7)	2546 (33.4)	1262 (17.2)	248 (4.5)		
Healthy level	17,783 (70.2)	1378 (28.3)	5068 (66.6)	6080 (82.8)	5257 (95.5)		
Physical activity (n)%						1.394	<.001
Unhealthy level	14,409 (56.9)	4617 (94.9)	5895 (77.4)	3323 (45.3)	574 (10.4)		
Healthy level	10,916 (43.1)	247 (5.1)	1719 (22.6)	4019 (54.7)	4931 (89.6)		
Waist (n)%						0.699	<.001
Unhealthy level	18,741 (74.0)	4567 (93.9)	6499 (85.4)	5374 (73.2)	2301 (41.8)		
Healthy level	6584 (26.0)	297 (6.1)	1115 (14.6)	1968 (26.8)	3204 (58.2)		
Diet (n)%						1.152	<.001
Unhealthy level	15,402 (60.8)	4649 (95.6)	5935 (77.9)	3778 (51.5)	1040 (18.9)		
Healthy level	9923 (39.2)	215 (4.4)	1679 (22.1)	3564 (48.5)	4465 (81.1)		
Hypertension (n)%						0.027	.027
No	349 (1.4)	67 (1.4)	87 (1.1)	98 (1.3)	97 (1.8)		
Yes	24,976 (98.6)	4797 (98.6)	7527 (98.9)	7244 (98.7)	5408 (98.2)		
Diabetes (n)%						0.252	<.001
No	21,393 (84.5)	3731 (76.7)	6181 (81.2)	6389 (87.0)	5092 (92.5)		
Yes	3932 (15.5)	1133 (23.3)	1433 (18.8)	953 (13.0)	413 (7.5)		
RA (n)%						0.137	<.001
No	24,089(95.1)	4500 (92.5)	7172 (94.2)	7036 (95.8)	5381 (97.7)		
Yes	1236 (4.9)	364 (7.5)	442 (5.8)	306 (4.2)	124 (2.3)		

HEI = healthy eating index, PIR = poverty income ratio, RA = rheumatoid arthritis, SMD = standardized mean difference.

### 3.2. Univariate logistic regression analysis

As illustrated in Table 4, the analysis indicates that female participants, nonwhite, and individuals with diabetes exhibit an elevated odds of having RA (OR > 1, *P* < .05). Conversely, participants who have attained at least a high school education, possess a PIR of ≥3.5, current nonsmoking, engage in low-to-moderate alcohol drinking, maintain adequate physical activity levels, and uphold a healthy waist circumference demonstrate an association with a lower likelihood of RA (OR < 1, *P* < .05).

**Table 4:** Weighted univariate logistic regression analysis of RA.

Variable	OR 95% CI	*P*-value
Gender		
Male	Ref	Ref
Female	1.36 (1.16–1.58)	<.001
Race		
Non-Hispanic White	Ref	Ref
Others	1.19 (1.02–1.39)	.026
Education		
Under high school	Ref	Ref
High school	0.79 (0.65–0.96)	.02
Above high school	0.49 (0.41–0.59)	.02
PIR		
<3.5	Ref	Ref
≥3.5	0.56 (0.46–0.68)	<.001
Smoke		
Unhealthy	Ref	Ref
Healthy	0.65 (0.55–0.76)	<.001
Drink		
Unhealthy	Ref	Ref
Healthy	0.57 (0.49–0.67)	<.001
Physical activity		
Unhealthy	Ref	Ref
Healthy	0.54 (0.47–0.63)	<.001
Waist circumference		
Unhealthy	Ref	Ref
Healthy	0.47 (0.38–0.59)	<.001
HEI		
Unhealthy	Ref	Ref
Healthy	0.92 (0.77–1.08)	.304
Hypertension		
No	Ref	Ref
Yes	1.21 (0.75–1.94)	.437
Diabetes		
No	Ref	Ref
Yes	2.41 (2.03–2.87)	<.001

CI = confidence interval, HEI = healthy eating index, OR = odds ratio, PIR = poverty income ratio.

### 3.3. The association between healthy lifestyle and rheumatoid arthritis

In an effort to explore the association between a comprehensive healthy lifestyle and RA, a multivariable weighted Logistic regression analysis was carried out. In the crude model, by comparing the participants with a healthy lifestyle score of 4 to 5 to those with a score of 0 to 1, the OR for RA was 0.266 (95% CI: 0.199–0.354). After adjusting for all covariates, the odds of having RA among adults with 4 to 5 healthy lifestyle factors were decreased by 58.3% compared to that among adults with 0 to 1 healthy lifestyle factors, with an OR of 0.417 (95% CI: 0.304–0.573). Additionally, for each additional healthy lifestyle factor, the odds of having RA was reduced by 20.8%, with an OR of 0.792 (95% CI: 0.735–0.853; Table 5).

**Table 5:** Association between healthy lifestyle and RA.

Score	Crude	*P*-value	Model 1	*P*-value	Model 2	*P*-value
Score	0.698 (0.654–0.746)	<.001	0.731 (0.681–0.784)	<.001	0.792 (0.735–0.853)	<.001
Group by number of healthy lifestyle factors						
0–1	Ref		Ref		Ref	
2	0.742 (0.593–0.929)	.010	0.766 (0.608–0.965)	.024	0.859 (0.683–1.081)	.192
3	0.535 (0.426–0.672)	<.001	0.582 (0.460–0.736)	<.001	0.700 (0.552–0.889)	.004
4–5	0.266 (0.199–0.354)	<.001	0.314 (0.234–0.423)	<.001	0.417 (0.304–0.573)	<.001

RA = rheumatoid arthritis.

### 3.4. Subgroup analyses and interaction test

To examine whether the association between the healthy lifestyle score and the odds of having RA varies among subgroups classified by age, gender, race, PIR, educational attainment, hypertension, and diabetes, we performed subgroup analyses and tests of interaction (Table 6). The tests of interaction indicated that the interaction among different age groups was statistically significant (*P* < .001), but a negative association between the healthy lifestyle and RA was observed in all age subgroups. No interaction was identified in the other subgroups (*P* > .05).

**Table 6:** Subgroup analysis and interaction test of the relationship between healthy lifestyle score and RA.

Level	OR (95% CI)	*P* value	*P* for interaction
Gender			.306
Female	0.78 (0.70, 0.86)	<.001	
Male	0.82 (0.73, 0.92)	<.001	
Race			.524
Non-Hispanic White	0.81 (0.73, 0.89)	<.001	
Others	0.77 (0.70, 0.86)	<.001	
Education			.352
Above high school	0.77 (0.70, 0.84)	<.001	
High school	0.85 (0.73, 0.98)	.03	
Under high school	0.80 (0.69, 0.93)	.004	
PIR			.846
<3.5	0.79 (0.73, 0.86)	<.001	
≥3.5	0.81 (0.72, 0.92)	.001	
Age			<.001
<50	0.67 (0.58, 0.76)	<.001	
≥50	0.88 (0.81, 0.95)	.002	
Hypertension			.637
No	0.92 (0.56, 1.53)	.75	
Yes	0.79 (0.73, 0.85)	<.001	
Diabetes			.045
No	0.77 (0.71, 0.83)	<.001	
Yes	0.90 (0.79, 1.03)	.12	

CI = confidence interval, OR = odds ratio, PIR = poverty income ratio, RA = rheumatoid arthritis.

### 3.5. Sensitivity analyses

**Figure 2.**
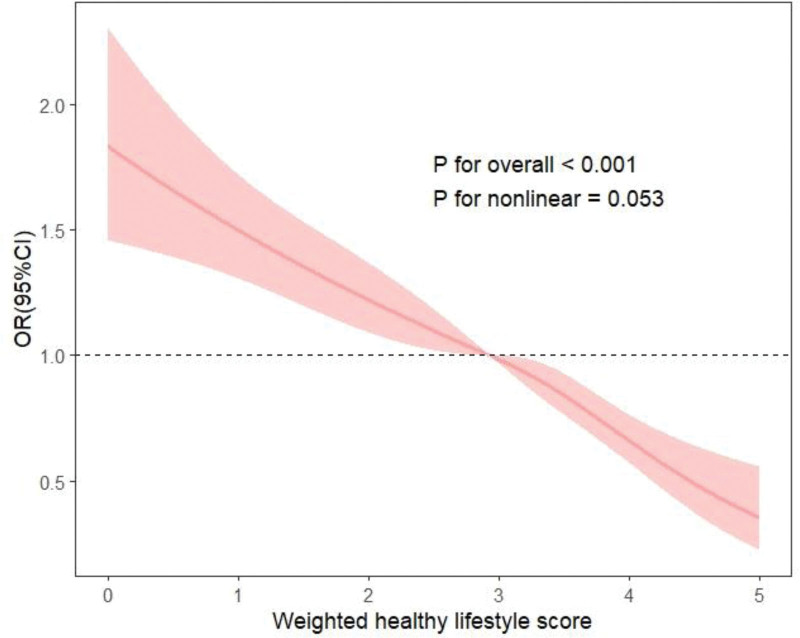
The RCS curve of weighted healthy lifestyle and RA incidence risk. RA = rheumatoid arthritis, RCS = restricted cubic splines.

Firstly, we analyzed the relationship between each individual healthy lifestyle and RA. It was found that current nonsmoking, low-to-moderate alcohol drinking, and adequate physical activity were all associated with a lower likelihood of RA (OR < 1, *P* < .05), yet the influence of healthy diet on RA was not significant (Table 7). When one of the current nonsmoking, low-to-moderate alcohol drinking, adequate physical activity, optimal waist circumference, and healthy diet was removed from the score respectively, the association between the score of 4 healthy lifestyle factors and RA weakened. The ORs (95% CI) comparing the 3 to 4 and 0 to 1 groups of healthy lifestyle factors were 0.560 (0.428–0.734), 0.541 (0.420–0.697), 0.537 (0.419–0.688), 0.576 (0.464–0.715), and 0.494 (0.399–0.612) respectively (Table 8). We further calculated the *E*-values (Table 9), and the *E*-value for the 4 to 5 group of the lifestyle score was 4.23, suggesting that a very strong confounding factor would be required to negate the negative association identified in our study. Finally, we constructed a weighted healthy lifestyle score to better reflect the effect of each healthy lifestyle factor on the outcome. As presented in Table 10, current nonsmoking made the greatest contribution to the weighted healthy lifestyle score (weighted β = 0.42), followed by optimal waist circumference (weighted β = 0.25), adequate physical activity (weighted β = 0.16), low-to moderate alcohol drinking (weighted β = 0.13), and healthy diet (weighted β = 0.03). After adjusting for all covariates, adults in the highest quartile of the weighted healthy lifestyle score had a 63.5% lower likelihood of RA compared to those in the lowest quartile, with an OR of 0.365 (0.253–0.527; Table 11). Furthermore, we plotted the restricted cubic spline graph of the relationship between the weighted healthy lifestyle score and RA to describe the dose–response relationship between the weighted healthy lifestyle score and the odds of RA (*P* for overall < .001, *P*-for nonlinear = .053; see Fig. 2). It can be observed that there is a linear relationship between the weighted healthy lifestyle score and the odds of RA, and the odds of RA continues to decrease with an increase in the weighted healthy lifestyle score.

**Table 7:** Weighted logistic regression analyses of each healthy lifestyle and RA.

Healthy lifestyle factor	OR (95% CI)	*P*-value
Current nonsmoking	0.628 (0.532–0.741)	<.001
Low-to-moderate drinking	0.779 (0.659–0.92)	.004
Adequate physical activity	0.738 (0.629–0.866)	<.001
Optimal waist circumference	0.687 (0.544–0.866)	.002
Healthy diet	0.901 (0.752–1.078)	.252

CI = confidence interval, OR = odds ratio, RA = rheumatoid arthritis.

**Table 8:** The relationship between adhering to the 4 healthy lifestyle habits and RA.

Score excluding 1 factor	0–1	*P*-value	2	*P*-value	3–4	*P*-value
Score excluding smoking	Ref	–	0.878 (0.729–1.058)	.170	0.560 (0.428–0.734)	<.001
Score excluding drinking	Ref	–	0.888 (0.732–1.077)	.225	0.541 (0.420–0.697)	<.001
Score excluding physical activity	Ref	–	0.684 (0.567–0.824)	<.001	0.537 (0.419–0.688)	<.001
Score excluding waist circumference	Ref	–	0.906 (0.736–1.115)	.349	0.576 (0.464–0.715)	<.001
Score excluding HEI	Ref	–	0.783 (0.651–0.941)	.010	0.494 (0.399–0.612)	<.001

HEI = healthy eating index, RA = rheumatoid arthritis.

**Table 9:** The *E*-value of the relationship between healthy lifestyle and RA.

Variable	OR (95% CI)	*E*-value
0–1 healthy lifestyle factors	Ref	–
2 healthy lifestyle factors	0.859 (0.683–1.081)	1.60
3 healthy lifestyle factors	0.700 (0.552–0.889)	2.21
4–5 healthy lifestyle factors	0.417 (0.304–0.573)	4.23
Each additional healthy lifestyle factor	0.792 (0.735–0.853)	1.84

CI = confidence interval, OR = odds ratio, RA = rheumatoid arthritis.

**Table 10:** The coefficients in the weighted logistic regression model.

Healthy lifestyle factor	Weighted β coefficient
Tobacco smoking (healthy vs unhealthy)	0.42
Alcohol drinking (healthy vs unhealthy)	0.13
Physical activity (healthy vs unhealthy)	0.16
Waist circumference (healthy vs unhealthy)	0.25
Diet (healthy vs unhealthy)	0.03

**Table 11:** The relationship between weighted healthy lifestyle score and RA.

	Crude	*P*-value	Model 1	*P*-value	Model 2	*P*-value
Weighted healthy lifestyle score	0.720 (0.683–0.759)	<.001	0.732 (0.690–0.777)	<.001	0.784 (0.737–0.833)	<.001
Group by quartiles of weighted healthy lifestyle score						
Q1	Ref		Ref		Ref	
Q2	0.767 (0.641–0.918)	.004	0.685 (0.568–0.825)	<.001	0.771 (0.643–0.924)	.005
Q3	0.473 (0.386–0.579)	<.001	0.455 (0.370–0.560)	<.001	0.559 (0.450–0.695)	<.001
Q4	0.231 (0.163–0.328)	<.001	0.289 (0.203–0.412)	<.001	0.365 (0.253–0.527)	<.001

RA = rheumatoid arthritis.

## 4. Discussion

This study ultimately incorporated 25,325 participants from the NHANES 1999–2018 cohort for analysis, encompassing 13,684 males and 11,641 females. Among them, 1236 patients were diagnosed with RA. A more healthful lifestyle was associated with a lower likelihood of RA, and this relationship remained stable in subgroups of different demographic characteristics and those with hypertension, diabetes, and other conditions. After removing one of the 5 lifestyle factors, this relationship attenuated. Weighted scoring could better reflect the association between a healthy lifestyle and RA. Furthermore, in the subgroup analysis, we found that after adjusting for all other covariates, there was an interaction between age and the healthy lifestyle score. In both the subgroups of age <50 and age ≥50, the OR values were all <1, indicating that the odds of RA was negatively associated with the healthy lifestyle score in both age groups, which was consistent with the results of the multivariate model. The OR value was smaller in the subgroup of age <50, suggesting that maintaining a healthy lifestyle might bring greater benefits to people under the age of 50.

Smoking may decrease the risk of RA onset. Smoking is related to extensive immune abnormalities, including alterations in T cell function, decreased numbers of NK cells, compromised humoral immunity, and elevated levels of inflammatory markers such as interleukin-6 (IL-6) and C-reactive protein. Its role in the pathogenesis of RA remains unclear and it may exert an effect in the early stage of immune dysregulation.^[37]^ This high-level systemic inflammatory response induced by tobacco is regarded as playing a crucial role in the pathogenesis of RA, and simultaneously, the imbalance of such cytokines may be associated with the poor treatment response of RA patients.^[38]^ Anti-cyclic citrullinated peptide antibody is a significant autoantibody in RA. Citrullinated proteins were present in the alveolar lavage fluid of smokers but not in that of nonsmokers, compared with nonsmokers.^[39]^

The Healthy Eating Index (HEI) serves as an indicator for evaluating dietary quality. The HEI has consistently been based on the quantity of food per 1000 kcal rather than absolute amounts and relies on a set of common standards applicable to both individuals and the environment. The HEI promotes adequate consumption of unsaturated fatty acids, a whole grain diet, and low added sugars. It has been well-demonstrated in in vitro studies and animal models that dietary unsaturated fatty acids exert effects on both the innate and adaptive immune systems, involving multiple immune cells and cytokines, which can lower the incidence and disease progression of RA, and reduce the duration of joint pain and morning stiffness.^[40]^ A whole grain diet can reduce systemic inflammatory responses and body weight compared with a refined grain diet.^[41]^ Abundant dietary sugar can enhance T-cell-induced inflammation. In conclusion, a healthy diet might lower the risk of developing RA by reducing systemic inflammatory responses and maintaining the balance of the immune system.^[42]^ In conclusion, a healthy diet is likely to reduce the risk of RA by mitigating systemic inflammatory responses and maintaining the equilibrium of the immune system. However, we found that after adjusting for all covariates, the association between HEI-2015 and RA was not significant. HEI takes into account all aspects of dietary quality but fails to fully consider the role of anti-inflammatory foods, which may have a stronger association with RA.^[43]^ A study indicates that some dietary components in the HEI are positively associated with the risk of developing RA, but the others are not significant.^[44]^ This requires further confirmation by subsequent prospective studies. We have noted that diet may interact with other lifestyle factors. A study has demonstrated that there is an inverse relationship between the intensity of tobacco consumption and the overall dietary quality,^[45]^ while alcohol consumption may influence dietary patterns by altering dietary behaviors.^[46]^ A high-quality diet contributes to maintaining an appropriate body weight. Exercise may reduce emotional eating and promote changes in dietary quality.^[47]^ Therefore, HEI is still retained in healthy lifestyle factors.

Physical activity can result in a marked elevation of T-reg cells, a decrease in immunoglobulin secretion, and a reduction in the generation of Th 1 cells. Furthermore, physical activity can also induce an anti-inflammatory response by facilitating the release of myogenic IL-6, which promotes the secretion of interleukin-10 and interleukin-1 receptor antagonist.^[48]^ Physical activity is the principal modifiable risk factor for obesity, while fat is the largest endocrine organ in the human body, and body fat seems to be the major determinant of inflammation.^[49]^ The indicator commonly employed to identify the presence and severity of excessive fat in adult bodies is BMI, yet BMI underestimates the influence of central obesity on health. Compared with BMI, waist circumference delineates fat distribution and has a stronger association with visceral fat, and can more robustly predict metabolic syndrome, cardiovascular diseases, and the like.^[50,51]^ A meta-analysis demonstrates that for each 10 cm increment in waist circumference, the risk of contracting RA rises by 13%.^[52]^

Interestingly, low to moderate alcohol consumption might reduce the risk of RA onset by lowering systemic inflammation levels.^[53]^ A meta-analysis reveals that alcohol consumption can significantly lower the risk of RA with positive cyclic citrullinated peptide.^[43]^ In animal experiments, alcohol inhibits the onset of collagen-induced inflammatory arthritis through downregulating leukocyte migration, upregulating testosterone secretion, and reducing nuclear factor-κB activation.^[44]^ Alcohol has also been demonstrated to exert anti-inflammatory effects in the human body through similar mechanisms, for instance, by reducing the production of inflammatory mediators in the monocyte-derived nuclear factor-κB pathway.^[54]^ However, heavy alcohol consumption can cause elevations in C-reactive protein, IL-6 and immunoglobulins, leading to an increase in the systemic inflammation level.^[55]^

Although much evidence indicates that lifestyle promotes the generation of inflammation, there are still studies suggesting that reverse causality may exist. The pain burden of RA patients may lead them to reduce physical activity. High disease activity can cause joint pain, swelling and morning stiffness, directly restricting the patients’ mobility.^[56]^ Furthermore, research has found that even after adjusting for disease activity, the physical activity of RA patients is still significantly lower than that of healthy individuals, suggesting that the structural damage of the disease itself (such as joint deformities) may have long-term effects.^[57]^ Pain is one of the main factors that lead RA patients to reduce their physical activities, especially joint pain and systemic fatigue, which significantly increase sedentary behavior.^[58]^ Objective measurements show that patients with severe pain have less time for moderate to vigorous physical activity each day.^[59,60]^ Long-lasting morning stiffness can prevent patients from participating in morning activities, and functional limitations (such as decreased grip strength and slower walking speed) further reduce the willingness to be active.^[59,61]^ Psychological factors can also lead to a reduction in physical activity. Approximately 30% to 50% of RA patients have a fear of exercise, worrying that it will aggravate joint damage or pain. This psychological barrier significantly reduces their participation in physical activities.^[62]^

The smoking rate among patients with chronic pain is significantly higher than that among people without pain (41.9% vs 26.6%), and the prevalence of chronic pain is also higher among smokers (32.9% among current smokers vs 17% among never smokers).^[63]^ This comorbidity phenomenon may stem from the short-term analgesic effect of nicotine being utilized by patients as a coping mechanism.^[64]^ The acute analgesic effect of alcohol may prompt patients to relieve pain by drinking. A study on soldiers found that chronic pain was significantly associated with drinking habits.^[65–67]^ Patients with chronic pain commonly exhibit “comfort eating” behavior, tending to choose high-sugar and high-fat comfort foods, which leads to an increase in the intake of saturated fatty acids.^[68,69]^ At the same time, patients with chronic pain commonly have a triad of “high saturated fat + high refined sugar + low fiber.”^[70,71]^ Chronic pain patients may also reduce their preparation of healthy diets due to limited mobility or emotional distress (such as depression), and instead rely on processed foods.^[72]^

Chronic inflammation and the medications used to treat RA may lead to an increase in patients’ waist circumference. Chronic inflammation in RA patients may cause muscle atrophy and visceral fat accumulation, resulting in “sarcopenic obesity,” and an increase in waist circumference is one of the indicators of increased visceral fat.^[73]^ Glucocorticoid drugs, such as prednisone, are known to promote central obesity. The changes in dietary and exercise behaviors caused by the chronic pain mentioned above may also indirectly contribute to the increase in waist circumference among RA patients.^[74]^

In conclusion, the score of healthy lifestyle is negatively associated with the odds of RA. On the 1 hand, maintaining a healthy lifestyle may help reduce the risk of developing RA. A large-scale observational study based on the UK Biobank database likewise supports our perspective.^[25]^ A considerable number of previous studies have been based on single-factor analysis, investigating the influence of altering a certain lifestyle on RA.^[22,75,76]^ On the other hand, our research is limited by cross-sectional studies and thus cannot reflect the causal relationship between the 2, to determine the causal relationship, additional cohort studies are needed.

Our research is the first 1 to explore the association between combined healthy lifestyles and RA based on large-sample data from the NHANES database. Additionally, as the NHANES database consists of multistage complex sampling data, we employed a weighted logistic regression model for analysis and adjusted for other covariates, rendering the conclusions drawn in this study more precise and reliable. Moreover, we also constructed a weighted comprehensive lifestyle score, which enables a better assessment of the odds of RA related to the current lifestyle.

However, our study has several limitations. Certain variables were derived from questionnaires and self-reports, which may introduce bias, although some studies have indicated that the accuracy rate of self-reported arthritis diagnosis is acceptable,^[77,78]^ the accuracy of the researchers’ own understanding of the disease and the standardization of the diagnostic process may both influence the study’s conclusions. Unfortunately, the NHANES database currently does not include serological markers related to RA, such as anti-cyclic citrullinated peptide, rheumatoid factor, and human leukocyte antigen DR4, which necessitates further investigation through large-scale prospective cohort studies. Similarly, the dietary information is derived from questionnaire-based recall, which is prone to recall bias. Despite the adjustment for a multitude of covariates, the potential for residual confounding cannot be entirely excluded. Furthermore, the cross-sectional design of this research emphasizes associations but does not establish causal relationships. Thus, cohort studies are essential for validating causality. Continued exploration of diagnostic and prognostic markers for RA by researchers is warranted. It is anticipated that this study will provide valuable scientific data references for future investigations.

## 5. Conclusions

In conclusion, our research provides novel insights into the relationship between RA and the healthy lifestyle score. Specifically, a negative association is observed between the healthy lifestyle score and RA. The existence of this association provides a new direction for subsequent research, enabling the exploration of the causal relationship between the 2. Compared with various single-factor lifestyle analyses, the healthy lifestyle score integrates multiple lifestyles and more comprehensively reflects the comprehensive exposure level of lifestyles. As a low-cost and easily assessable indicator, the healthy lifestyle score provides preliminary directional insights for public health practice.

## Acknowledgments

The authors thank all the participants and staff of the National Health and Nutrition Examination Survey and the National Center for Environmental Health for their valuable contributions.

## Author contributions

**Data curation:** Xuming Zhang.

**Methodology:** Shouyu Miao, Xuming Zhang.

**Software:** Xuming Zhang.

**Writing – original draft:** Shouyu Miao, Xuming Zhang.

**Writing – review & editing:** Xuming Zhang.
